# A guideline for screw fixation of coracoid process base fracture by 3D simulation

**DOI:** 10.1186/s13018-021-02203-0

**Published:** 2021-01-14

**Authors:** Zhongye Sun, Hao Li, Bei Wang, Jun Yan, Liren Han, Shizhang Han, Xiaofei Yang, Bei Zhao

**Affiliations:** 1 Department of Orthopaedics, Liaocheng People’s Hospital, 67 Dongchang West Road, Liaocheng, 252000 Shandong China; 2 Department of Imaging, Liaocheng Infectious Disease Hospital, Liaocheng, Shandong China

**Keywords:** Coracoid process base, Axial perspective, Digital measurement, Screw fixation

## Abstract

**Background:**

Fractures of the base of the coracoid process are relatively rare, but an increasing number of studies have reported using screws to fix coracoid process base fractures. This study was performed to simulate the surgical procedure and obtain the ideal diameter, length, insertion point and angle of the screw from a 3-D axial perspective in Chinese patients.

**Methods:**

We randomly collected right scapula computed tomography (CT) scans from 100 adults. DICOM-formatted CT scan images were imported into Mimics software. A 3D digital model of the right scapula was established. Two virtual cylinders representing two screws were placed from the top of the coracoid process to the neck of the scapula and across the base of the coracoid process to fix the base of the coracoid process. The largest secure diameters and lengths of the virtual screws were measured. The positions of the insertion points and the directions of the screws were also examined.

**Results:**

The screw insertion safe zone can exhibit an irregular fusiform shape according to the reconstructed scapula model. The mean maximum diameters of the medial and lateral screws were 7.08 ± 1.19 mm and 7.34 ± 1.11 mm, respectively. The mean maximum lengths of the medial and lateral screws were 43.11 ± 6.31 mm and 48.16 ± 6.94 mm, respectively. A screw insertion corridor with a diameter of at least 4.5 mm was found in all patients. We found sex-dependent differences in the mean maximum diameters and maximum lengths of the two screws. The positions of the two insertion points were statistically different across sexes.

**Conclusions:**

The study provides a valuable guideline for determining the largest secure corridor for two screws in fixing a fracture at the base of the coracoid process. For ideal screw placement, we suggest individualised preoperative 3D reconstruction simulations. Further biomechanical studies are needed to verify the function of the screws.

## Background

Fractures of the coracoid process base are rare, and current treatment guidelines remain unclear [1]. Ogawa et al. proposed a classification system for coracoid fractures based on the relationship between the fracture site and the coracoclavicular ligament. Type I fractures are located behind the CC ligaments, whereas type II fractures are anterior to it. They considered that type I fractures indeed require operation whenever the scapuloclavicular connection has been destroyed [2]. This is consistent with many reports in the literature [1–13]. Within these injuries, a fracture of the coracoid process base represents a severe form of the injury, and a variety of classifications have stressed the importance of recognition of this subtype. The definitive fixation for fracture of the coracoid process base is performed with 1 to 2 screws ± washers [2, 4–9, 11, 12, 14]. Hill et al. believed that a second screw may be used to supplement a single screw and has the benefit of controlling rotation of the fracture thus enhancing fixation against traction and rotational forces of the upper extremity. The coracoid process is in close proximity to major neurovascular structures, including the brachial plexus and the axillary artery and vein [15]. Knowledge of the correct location of the insertion point and screw direction is essential to avoid penetrating the joint and injuring neurovascular structures. In addition, the complex anatomy of the coracoid process and the ligaments and muscles attached to it make screw placement more difficult. Bhatia et al. described percutaneous coracoid base fixation using orthogonal biplanar fluoroscopic guidance techniques. Nevertheless, they noted that theoretical complications such as articular perforation, neurovascular injury and damage to coracoclavicular ligaments may emerge even when the surgery is performed by an experienced shoulder surgeon [12]. Kawasaki et al. reported a new screw fixation technique for coracoid base fractures under fluoroscopic guidance and considered anatomic information on the cross-sectional size of the coracoid base obtained in a computed tomography (CT) study [7].

Hill et al. described that if the fracture is not comminuted and occurs through the base, then a 3.5-mm lag screw is often needed for adequate stability. They used a screw length between 30 and 45 mm, with 15 degrees of medial angulation and 30–40 degrees of posterior angulation to ensure that the screw remained enclosed in the bone [6]. Many reports also used their fixation technique and found good postoperative outcomes. Although some open, mini-open and percutaneous techniques under fluoroscopic guidance have been reported previously [2, 4–9, 11, 12, 16], screw insertion into the neck of the scapula across the fracture of the coracoid base is difficult due to the complex shape of the scapula [17, 18].

Translational medicine has been widely studied for more than 10 years, and it solves the problem of how to fill the gap between basic sciences and clinical sciences [19]. This inspired us to explore how to use 3D simulation for orthopaedic procedures and obtain the ideal diameter, length, insertion point and angle of the screw. 3D simulation helps surgeons understand the important anatomical structures (nerves, vessels) and anatomical properties (length, angles, anatomical axis) [20, 21]. It has been widely used in orthopaedics such as tumour bone [22] and thermal necrosis [23].

At present, there are many studies on the application of CT data in various software programs for the fixation of screws in the treatment of different fractures [24–26]. In previous studies, only the length of the long and short axes at the thinnest part of the coracoid base in the axial CT plane was measured [7]. The purpose of the study was to specify the ideal insertion points, the largest secure diameters and lengths and the appropriate angles for the two screws from an axial perspective.

## Materials and methods

We retrospectively collected the right scapula CT scans of 100 adults who had undergone continuous slice CT scanning at the imaging research centre of our hospital between August 2018 and July 2020. Patients were excluded if they had scapula fractures, tumours, or severe deformities. This study was approved by the Institutional Review Board of our hospital, and patients’ informed consent was obtained. The mean age of the patients on whom the models were based was 47.96 ± 16.12 years (range 18–85 years).

**Fig. 1:**
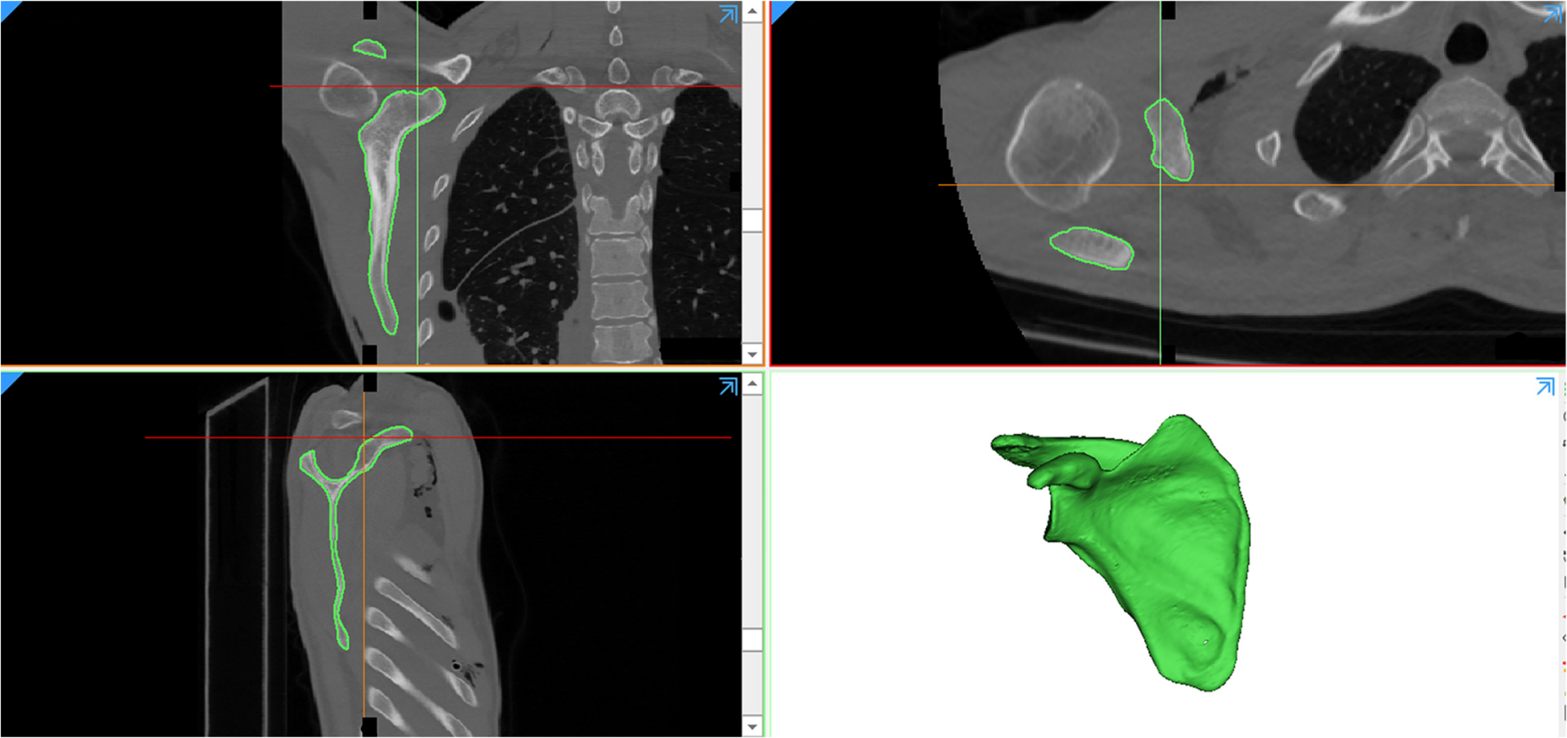
DICOM-formatted CT scan images were imported into Mimics software. Then, a right virtual scapula model was created by image segmentation, region growth and multiple slice editing

DICOM-formatted CT scan images of each patient were imported into Mimics software (21.0; Materialise, Leuven, Belgium). We removed the soft tissue by image segmentation, region growth and multiple slice editing (Fig. 1). A total of 100 right virtual scapula models were created.

**Fig. 2:**
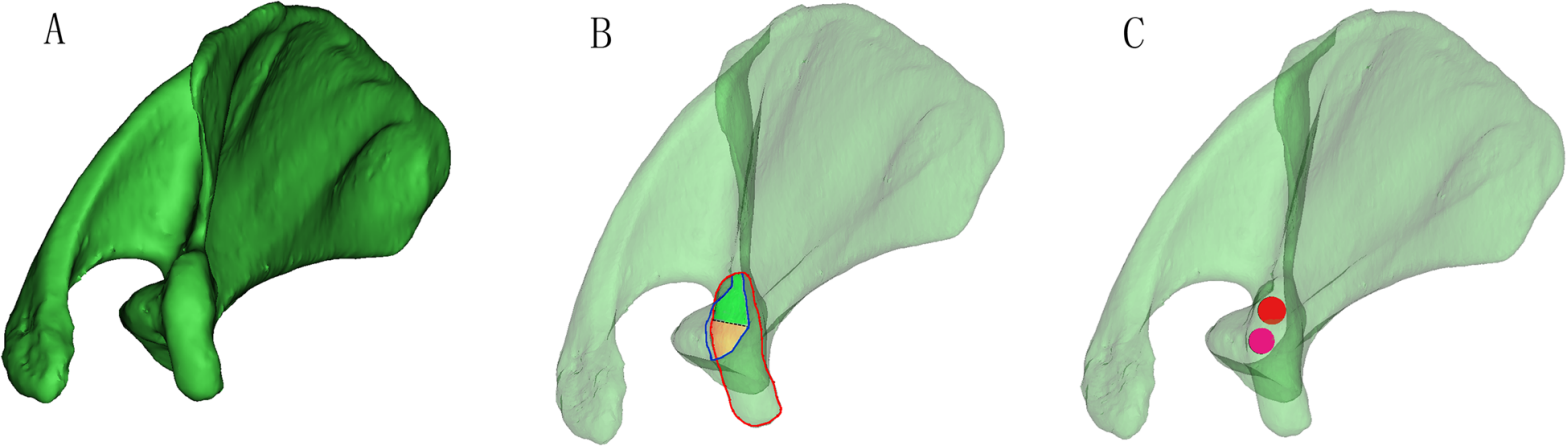
Find the largest screw path. **a** The 3D model was turned to the axial perspective to find the largest translucent area. **b** The red outline represents the top boundary of the horizontal part of the coracoid process, and the blue outline represents the boundary of the cross section of the base of the coracoid process. The green and orange areas represent the two screw paths. **c** Two virtual screws were inserted into the green and orange areas, respectively. Then, the diameters were increased progressively until they reached the borderline of the area (the red circle of the cylinder represents the largest medial screw, and the purple one represents the largest lateral screw)

**Fig. 3:**
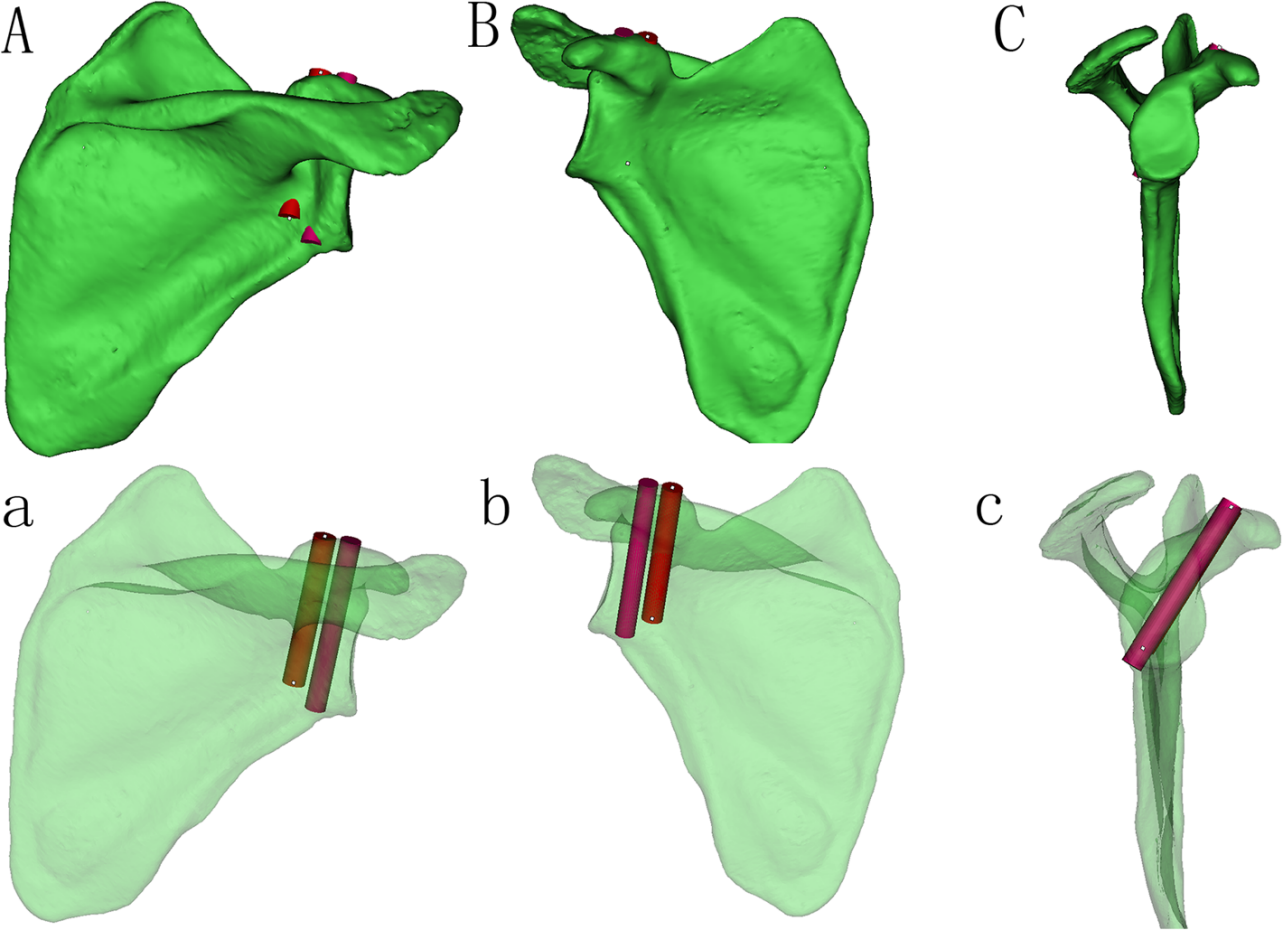
The position of the virtual screws were verified in the 3D model. A, B, C Observed from the posterior, anterior and lateral of the opaque 3D model, respectively. The screws had the largest lengths and diameters just penetrating the cortical bone. a, b, c Observed from the posterior, anterior and lateral of the translucent 3D model, respectively. Adjusted to the optimal lengths and diameters of the screws from the translucent 3D model

**Fig. 4:**
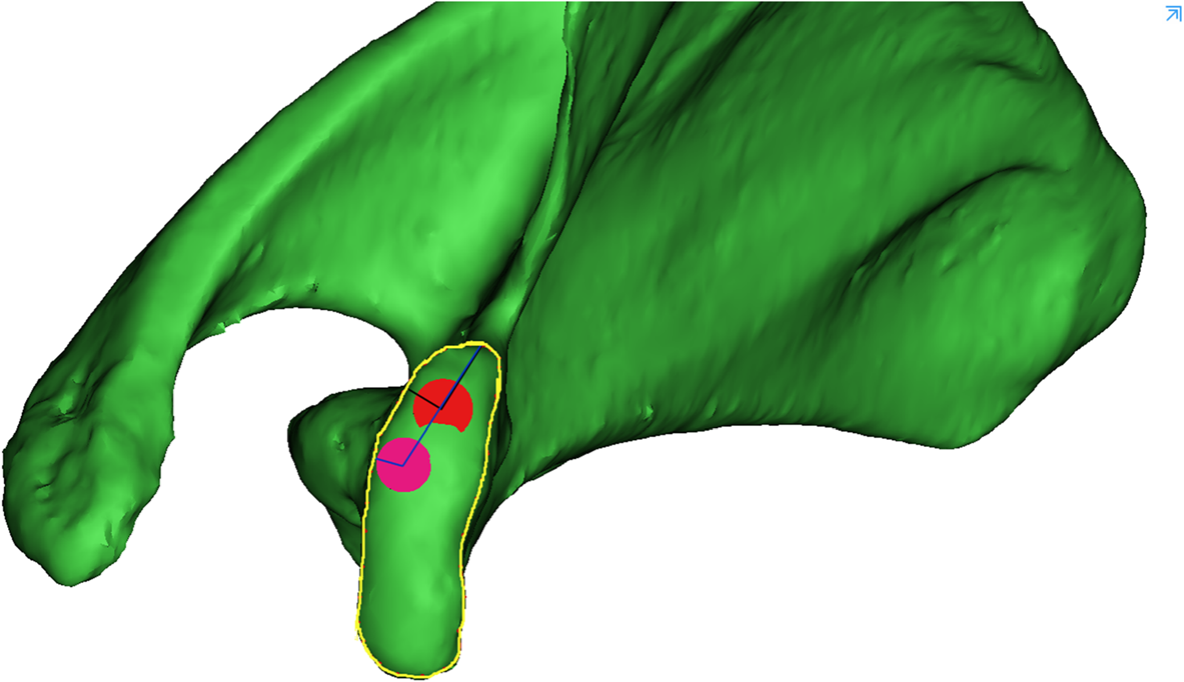
The measurement of distance L1, L2, L3 and L4. The yellow outline represents the boundary of the horizontal part of the coracoid process. The distances from the medial and lateral screw insertion points to the closest point of the coracoid were recorded as distance L1 and L3, respectively. The vertical distances from the medial and lateral screw insertion points to the posterior border line of the horizontal part of the coracoid were recorded as distance L2 and L4, respectively

**Fig. 5:**
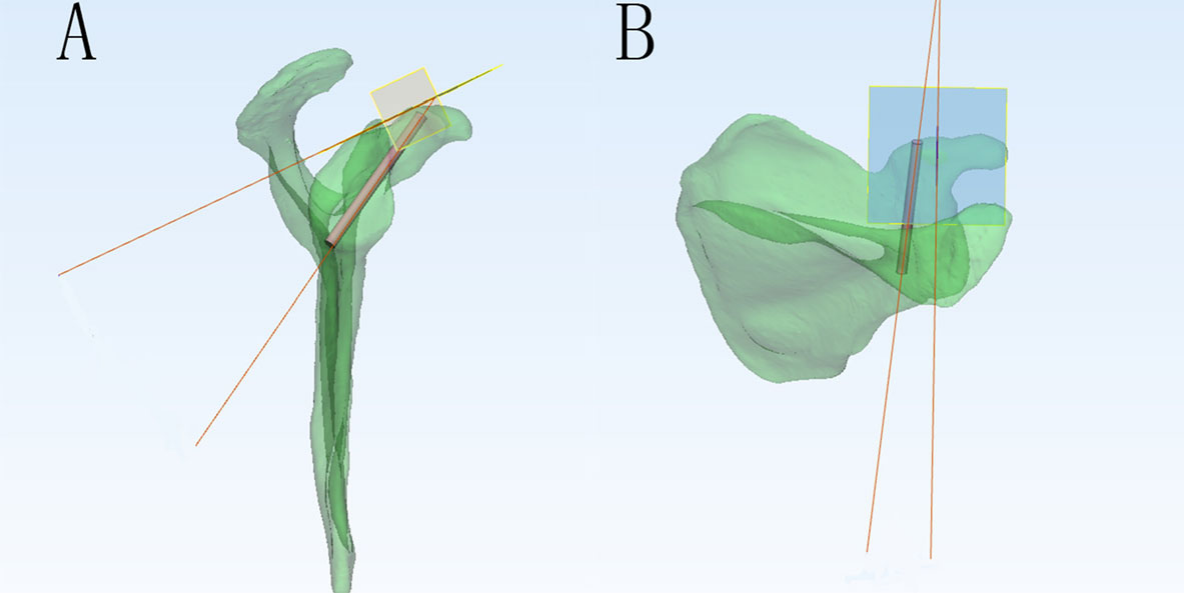
The measurement of angle *α* and *β*. **a** The slope of the upper edge of the posterior coracoid process was selected as the reference plane and called plane 1 (yellow plane). The anterior inclination angle between the screw and plane 1 was measured and recorded as angle *α*. **b** The other reference plane perpendicular to plane 1 was called plane 2 (blue plane). The medial inclination angle between the screw and plane 2 was measured and recorded as angle *β*

We reduced the transparency of the right scapula models and focused on the axial perspective view, which was parallel to the cross section of the base of the coracoid process from top to bottom (Fig. 2a). We observed and adjusted the position of the model to find the largest translucent area through the perspective view. Then, a translucent area with an irregular fusiform shape was clearly seen and divided into two basically equal parts to implant two screws (Fig. 2b). The red outline represents the top boundary of the horizontal part of the coracoid process, and the blue outline represents the boundary of the cross section of the base of the coracoid process. The green and orange areas represent the two screw paths. Two virtual cylinders representing the screws were placed into the translucent area. The diameter increased progressively, and the maximum diameter was defined when the cylinder did not penetrate the border of the area (Fig. 2c). We observed and adjusted the length of the screw to ensure that the screw just penetrated the posterior cortical bone (Fig. 3a-c). The diameters and lengths of the virtual screws were measured. To confirm the position of the screw, the distances from the insertion point to the closest point of the coracoid and the posterior border line of the horizontal part of the coracoid were measured. They were recorded as distances L1 and L2 for the medial screw (MS) and L3 and L4 for the lateral screw (LS) (Fig. 4). The slope of the upper edge of the posterior coracoid process was selected as the reference plane and called plane 1. The anterior inclination angle between the screw and plane 1 was measured and recorded as angle α (Fig. 5a). In addition, we defined another reference plane perpendicular to plane 1 called plane 2. The medial inclination angle between the screw and plane 2 was also measured and recorded as angle β (Fig. 5b).

The collected data were analysed by SPSS 25.0 statistical software. The experimental data are represented as the mean ± SD. *t* tests were used to compare the data. Statistical significance was accepted at *P* < 0.05.

## Results

The study subjects included 50 males and 50 females aged between 18 and 85 years old, with a mean age of 47.96 ± 16.12 years. As shown in Fig. 2b, the screw insertion safe zone can exhibit an irregular fusiform shape according to the reconstructed scapula model.

As shown in Tables 1 and 2, the mean maximum diameters of the medial and lateral screws were 7.08 ± 1.19 mm and 7.34 ± 1.11 mm, respectively. The mean maximum lengths of the medial and lateral screws were 43.11 ± 6.31 mm and 48.16 ± 6.94 mm, respectively. The mean distance L1 was 11.63 ± 2.87 mm, L2 was 7.50 ± 1.72 mm, L3 was 19.87 ± 2.76 mm and L4 was 4.88 ± 0.86 mm. For the data captured above, the intersex difference was significant (*P* < 0.05).

**Table 1:** Comparison between different genders: diameters of medial screws, lengths of medial screws, L1 and L2

Group	Diameter^#^ (mm)	Length^#^ (mm)	L1^#^ (mm)	L2^#^ (mm)
All (*n* = 100)	7.08 ± 1.19	43.11 ± 6.31	11.63 ± 2.87	7.50 ± 1.72
Male (*n* = 50)	7.89 ± 0.98	47.62 ± 4.29	12.36 ± 2.70	8.21 ± 1.68
Female (*n* = 50)	6.27 ± 0.76	38.60 ± 4.54	10.89 ± 2.87	6.80 ± 1.45
*t* value*	9.237	10.207	2.629	4.475
*P* value*	0.00	0.00	0.01	0.00

*t and *P* are the results of gender comparisons

^#^For the diameter, length, the distance of L1 and L2, intersex difference was significant (*P* < 0.05)

**Table 2:** Comparison between different genders: diameters of lateral screws, lengths of lateral screws, L3 and L4

Group	Diameter^#^ (mm)	Length^#^ (mm)	L3^#^ (mm)	L4^#^ (mm)
All (*n* = 100)	7.34 ± 1.11	48.16 ± 6.94	19.87 ± 2.76	4.88 ± 0.86
Male (*n* = 50)	8.06 ± 0.81	52.81 ± 5.40	21.08 ± 2.51	5.06 ± 0.70
Female (*n* = 50)	6.61 ± 0.87	43.52 ± 4.91	18.66 ± 2.47	4.69 ± 0.96
*t* value*	8.655	8.997	4.856	2.181
*P* value*	0.00	0.00	0.00	0.03

*t and *P* are the results of gender comparisons

^#^For the diameter, length, the distance of L3 and L4, intersex difference was significant (*P* < 0.05)

The mean angles *α* and *β* of the different sexes are also recorded in Table 3. The former was 16.40° ± 6.03° and the latter was 10.33° ± 7.39°. The results of angle β were statistically different between males and females (*P* < 0.05). However, angle α was not statistically different (*P* > 0.05).

**Table 3:** Comparison between different genders: angle *α* and *β*

Group	*α* (°)	*β* (°)^#^
All (*n* = 100)	16.40 ± 6.03	10.33 ± 7.39
Male (*n* = 50)	16.58 ± 5.78	8.39 ± 6.65
Female (*n* = 50)	16.21 ± 6.33	12.26 ± 7.64
*t* value*	0.304	– 2.696
*P* value*	0.762	0.008

*t and *P* are the results of gender comparisons

^#^For the angle *β*, intersex difference was significant (*P* < 0.05)

A screw insertion corridor with a diameter of at least 4.5 mm was found in everyone in our research.

## Discussion

In 1996, Ogawa et al. believed that a type 1 coracoid fracture was more unstable and required open reduction and internal fixation. They fixed unstable fractures of the coracoid base with a malleolar screw and washer [2]. Hill et al. described in detail the technique and clinical experience of screw fixation for fracture of the coracoid base. The difference between their study and that of Ogawa et al.’s is that another screw may be used to supplement the single screw and has the benefit of offering rotational control of the fracture, thus enhancing the fixation against traction and rotational forces of the upper extremity [6]. We share this view that the common fixation method for coracoid base fractures is to pass two parallel screws vertically through the fracture line. He provided details on the surgical approaches and nail placement angles, but the exact point of entry was not given. The angle and the diameter and length of the screw will change with different entry points.

There are a variety of screws used to fix coracoid process base fractures in a number of past reports [2, 4–9, 11, 12, 14]. The vast majority of them are cannulated cancellous screws of different sizes [2, 4, 5, 7–9, 11, 12, 14]. Hill et al. used cortical lag screws [6]. Ogawa et al. suggested using a 4.5-mm malleolar screw or a cannulated screw for both length and anchoring strength [27]. Beranger et al. reported on a CT scan-based coracoid process bone mineral density measurement [28]. They found that the bone mineral density of the coracoid process decreased significantly with age. To avoid coracoid process fracture during the Latarjet intervention, they recommended the use of partially threaded cancellous screws as the modality of fixation. The use of guide wire makes it easy to place a cannulated screw, and a cancellous screw can enhance the holding force in the bone. Therefore, we recommend the use of partially threaded cannulated cancellous screws.

Many anatomic and radiographic measurements of the coracoid process have been reported [17, 18, 29, 30]. Kawasaki et al. reported the cross-sectional size at the level of the coracoid base by a study on CT axial measurement of the coracoid base [7]. The measurement data on the coracoid base may be useful for safe screw fixation of coracoid base fractures.

The coracoid process is complex in structure and varies from person to person [17, 18]. Previous reports did not involve a large number of patients whose coracoid base fractures required screw fixation nor did they report the entry points and angles used or screw length and diameter statistics. There are few digital anatomical studies on its properties.

Mimics software has been widely used for 3D reconstruction in the development of digital orthopaedic technology. In our study, we applied the 3D method from the axial perspective as described in previous studies [24–26]. We observed and adjusted the position of the model to find the largest translucent area through the perspective view. Then, the translucent area, like an irregular fusiform shape, was divided into two basically equal parts to implant two screws. We increased the diameters of two virtual screws progressively and monitored the virtual screws in the views of the coronal plane, sagittal plane and horizontal plane, without violating the cortices and articular surface. The method used in our study not only saves manpower, materials and financial resources but can also be repeated and verified by test results with high reliability.

In our research, we recorded the exact points of entry. The distances from two screw insertion points to the closest point of the coracoid and the vertical distances from two screw insertion points to the posterior border line of the horizontal part of the coracoid were all observed in this study. There are significant sex differences. The data captured above are due to the obvious anatomic differences in scapula bones between females and males.

Many screws with a diameter of 3.5 mm or 4.5 mm have been reported for fixation of coracoid process base fractures [2, 4–9, 11, 12]. According to the information in our study, to avoid cortical breaches, the maximum diameter was 7.89 ± 0.98 mm (MS) and 8.06 ± 0.81 mm (LS) in males and 6.27 ± 0.76 mm (MS) and 6.61 ± 0.87 mm (LS) in females. Everyone possessed a corridor with a diameter of at least 4.5 mm. Nevertheless, due to individual and sex differences, the use of preoperative measurements and calculations by digital tools is recommended.

Hill et al. described the screws he used to fix the base fracture of the coracoid process as 30–45 cm [6]. In our study, we measured the maximum length of the screws just passing through the posterior cortex of the scapula. The length of the medial screw was 47.62 ± 4.29 mm in males and 38.60 ± 4.54 mm in females. The length of the lateral screw was 52.81 ± 5.40 mm in males and 43.52 ± 4.91 mm in females. It turns out that we can actually choose a slightly longer screw.

On the basis of determining the best diameter and length of the screw, the insertion point and direction are two important factors affecting the safe placement of screws. Previous reports have not given the exact points of entry. Different from previous studies, we found that the optimised insertion points are 12.36 ± 2.70 mm (MS) and 21.08 ± 2.51 mm (LS) away from the closest point in males and 10.89 ± 2.87 mm (MS) and 18.66 ± 2.47 mm (LS) in females; simultaneously, they are 8.21 ± 1.68 mm (MS) and 5.06 ± 0.70 mm (LS) away from the posterior line in males and 6.80 ± 1.45 mm (MS) and 4.69 ± 0.96 mm (LS) in females. The anatomic landmark of the closest point and the posterior line of the coracoid process can be easily palpated and identified, so they can be used as effective references intraoperatively.

Hill et al. used a screw with 15° of medial angulation and 30–40° of posterior angulation to ensure that the screw remained enclosed in the bone [6]. Because of the difference in the reference plane, the results cannot be compared. We believe that the exact coordinates of the measurement angle are not given in the previous research report, which leads to the imprecision of the measured angle. The angle of measurement will vary depending on the position of the scapula. In our study, we measured a significant gender difference in angle *β*.

Trikt et al. reported a useful fluoroscopic view based on simple landmarks for fixation of fractures of the coracoid base [31]. Their approach is similar to ours, and an optimal trajectory for the placement of screws in the base fracture of the coracoid process was also obtained. However, there are many differences. Instead of using cadaver studies, we used the CT scans of 100 patients in our hospital. In our study, the sample size was larger. In our study, two screws were recommended for the fixation of coracoid process base fractures, so the parameters of two screws were measured rather than one screw. They developed a fluoroscopic view as a useful radiographic technique for orthopaedic surgeons to fix fractures of the coracoid base. We measured the screw parameters for guidance for screw fixation of coracoid process base fractures, and the sex difference in screw parameters was analysed statistically.

The parameters of the two screws may provide the surgeon with appropriate information on safe screw placement for the treatment of coracoid base fractures. The large standard deviation of our results indicates great differences among individuals. As a result, preoperative planning should be implemented in detail for each patient. 3D reconstruction and simulated screw placement techniques with digital software before surgery are valuable.

There are some limitations to this study. We only analysed the data according to sex, not according to different age groups. In addition, we only studied the scapula of Chinese people, who have different skeletal shapes than European and American individuals. Software tools cannot replace experimental testing, but they provide a valuable and rapidly evolving option [32]. Moreover, more biomechanical studies and related clinical research should be performed.

## Conclusion

We indicate valuable guidelines for screw fixation of coracoid process base fractures by 3D simulation. The ideal screw position and the size of the screws can be determined in 3D models by digital software. Further biomechanical studies on different screw-fixed coracoid process base fractures are needed to verify the strength and effect. Further clinical studies are needed to validate the protocol and determine the accuracy of this technique in a clinical setting.

## Data Availability

The datasets generated and analysed during the current study are available from the corresponding author on reasonable request.

## Abbreviations

3-D: Three-dimensional
CT: Computed tomography
DICOM: Digital Imaging and Communication in Medicine
Mimics: Materialise’s Interactive Medical Image Control System
SPSS: Statistical Package for the Social Sciences
SD: Standard deviation
CC: The coracoclavicular
MS: The medial screw
LS: The lateral screw
